# Design and psychometric evaluation of schools’ resilience tool in Emergencies and disasters: A mixed-method

**DOI:** 10.1371/journal.pone.0253906

**Published:** 2021-07-22

**Authors:** Samaneh Mirzaei, Leila Mohammadinia, KHadijeh Nasiriani, Abbas Ali Dehghani Tafti, Zohreh Rahaei, Hossein Falahzade, Hamid Reza Amiri, Hamid Sharif Nia, Mohammad Hossein Dehghani

**Affiliations:** 1 Health in Disasters and Emergencies, Student Research Committee, Shahid Rahnemoun Hospital, Shahid Sadoughi University of Medical Sciences, Yazd, Iran; 2 Department of Health in Disasters and Emergencies, Health and Human Resources Research Center, School of Management and Information Sciences, Shiraz University of Medical Sciences, Shiraz, Iran; 3 Department of Health Services Management, Faculty of Management and Medical Informatics, Tabriz University of Medical Sciences, Tabriz, Iran; 4 Research Center for Nursing and Midwifery Care, Department of Nursing, Nursing and Midwifery School, Shahid Sadoughi University of Medical Sciences, Yazd, Iran; 5 Department of Health in Emergencies and Disasters, School of Public Health, Shahid Sadoughi University of Medical Sciences, Yazd, Iran; 6 Department of Health Education, School of Public Health, Shahid Sadoughi University of Medical Sciences, Yazd, Iran; 7 Department of Biostatistics and Epidemiology, School of Public Health, Shahid Sadoughi University of Medical Sciences, Yazd, Iran; 8 Department of Civil Engineering, Yazd Branch, Islamic Azad University, Yazd, Iran; 9 Department of Nursing Mazandaran University of Medical Science, Sari, Iran; 10 Department of Anesthesiology and Critical Care, Shahid Rahnemoun Hospital, Shahid Sadoughi University of Medical Sciences, Yazd, Iran; University of Bologna, ITALY

## Abstract

**Background:**

In addition to their educational role, resilient schools have a good capacity in response to disasters. Due to the large student population, the schools can be a safe and secure environment during disasters, in addition to maintaining their performance after. Given the role and importance of the schools, the impact of culture and environment on resilience, without any indigenous and comprehensive tool for measuring the resilience in Iran, the study aimed to design and psychometrically evaluate the measurement tools.

**Method:**

This study was conducted using a mixed-method sequential explanatory approach. The research was conducted in two main phases of production on items based on hybrid model and the psychometric evaluation of the tool. The second phase included validity (formal, content and construction) and reliability (multiplex internal similarity, consistency and reliability).

**Result:**

The integration of systematic and qualitative steps resulted in entering 91 items into the pool of items. After formal and content validity, 73 items remained and 44 were omitted in exploratory factor analysis. A questionnaire with 5 factors explained 52.08% of total variance. Finally, after the confirmatory factor analysis, the questionnaire was extracted with 29 questions and 5 factors including "functional", "architectural", "equipment", "education" and "safety". Internal similarity and stability in all factors were evaluated as good.

**Conclusion:**

The result showed that the 29-item questionnaire of school resilience in emergencies and disasters is valid and reliable, that can be used to evaluate school resilience. On the other hand, the questionnaire on assessment of school resilience in disasters enables intervention to improve its capacity.

## Background

Resilience is defined as a process of successful adaptability despite threatening conditions [1], The concept of resilience has recently experienced surging popularity [2]. This concept is applicable to many fields, from mechanics to a broad kind of social sciences [3]. Resilience has become an important concept in the fields of disaster management [4]. Resilience involves both physical and social systems [5] and includes three dimensions of persistence, improvement and self-reliance [6]. So, a resilient community is able to respond to change or stress in a positive way. It can also maintain its core functions as a whole despite the tensions that exist [7]. Therefore, the role of planners and policymakers in the formation of resilience is very important [8]. Identifying resilience indicators (economic, social, institutional, environmental or infrastructural) can provide a useful method for examining different locations and comparing between and within each area after creation [7]. The Hyogo (2005–2015) [9] and Sendai (2015–2030) documents have also highlighted the importance of resilience in reducing disaster risk and have been identified as important cases for increasing preparedness [10–13].

Schools as educational environments can play an effective role in reducing disaster risk and increasing resiliency [14]. On the other hand, they are used as shelters for care and relief after disasters [15, 16]. In general, resilient schools have minimal vulnerability to disasters. And if they cope with minimal casualties and damage, they achieve effective performance in the shortest time possible [17]. Various components, such as structural, non-structural, functional process and facilities are effective factors in increasing readiness and resilience of schools in disasters and emergencies [18–21]. On the other hand, components such as structural and non-structural improvements, enhancing organizational coordination and interactions, improving training and process are known as operational strategies for establishing resilient schools [22].

In a systematic sense, these factors can influence the process of school education. As a result, if schools are prepared to required standards, they can improve their performance. Therefore, to achieve this in schools, it is necessary to observe these issues at all levels from preschool to high school (state and non-state) [23]. Schools, as the most important sources of social and economic development, should take advantage of the involvement of organizations, institutions and people in the provision of student health, prevention and control of emergencies in their public education. It seems that further coordination between the University of Medical Sciences, Health Services and the Education Authority in full implementation of school health regulations, revising design, construction and equipping of schools, training of health coaches and school principals, promoting safety and hygiene culture and increasing school health per capita can be effective in improving environmental health and safety in schools [24].

As students and teachers spend a lot of time in schools, the vulnerability and resilience of the schools in disasters and disasters should be considered [25]. The impact of emergencies and disasters in schools can be damaged to their construction and infrastructure or psychological effects on students and staff [26, 27]; and this has negative effects on the educational process and performance of schools [16, 28]. As a result, in resilient and disaster-prone schools, training staff and students in disasters and emergencies, hazard identification, coherent risk reduction program, provision of disaster-relief items, mutual and effective communication; cooperation with the family of students and emergency services organizations are considered [17]. Providing programs and supplies for safe and accessible schools, involvement and leadership of schools in disaster risk reduction programs, decision making processes and joint effort with other stakeholders towards risk mitigation within the school promotes effective participation [29].

The evaluation of school resilience should be based on appropriate tools such as school resilience assessment, that examines school resilience in terms of five-dimensional climate: physical conditions; human resources; fundamental issues; external relations and natural conditions, which included 75 items. The use of this tool displays the importance of strengthening the relationship between school and community and enhancing the involvement of different stakeholders in planning process [18]. In another tool provided by Dwiningrum et al., in 2017 [29] to measure school resilience and understand students’ teachings to create school resilience, six variables, such as enhancing communication, defining clear boundaries, teaching life skills, caring and support, setting and communicating high expectations, and providing opportunities for meaningful participation are considered as important aspects in school resilience to reduce social harm. In this study, the reduction of environmental risk and building resilience in the same were also considered [30].

Periodic evaluation of schools during disaster recovery is essential in order to adopt appropriate activities and policies, and apply the results to better management [18]. Resilience has broader dimension than preparedness, safety or and response planning of disasters that need to be addressed to increase schools’ ability to maintain proper performance and response after disasters, and tools that address all aspects of resilience not found in current study. Moreover, review of national studies also showed that there is no tool for assessing school resiliency in accordance with Iran’s native situation which indicates necessity of developing a comprehensive tool for measuring the resiliency in disasters and emergencies based on Iranian situation [31].

Since schools can be considered as safe and secure environments in the face of disasters, and post-disaster reconstruction phase, one of the criteria of returning community to normal conditions is determined by reopening the schools [20, 32]. As culture and environment influence resilience, it varies from one society to another, and there are temporal, geographic and cultural differences among societies. Therefore, it is necessary to provide appropriate native tools for assessing and evaluating effective factors on school resiliency, and to make available to trustees such as the Education Authority [33]. This study, therefore, aimed to design and validate school resilience measurement tools in disasters and emergencies.

## Methods

### Study design

**Fig 1:**
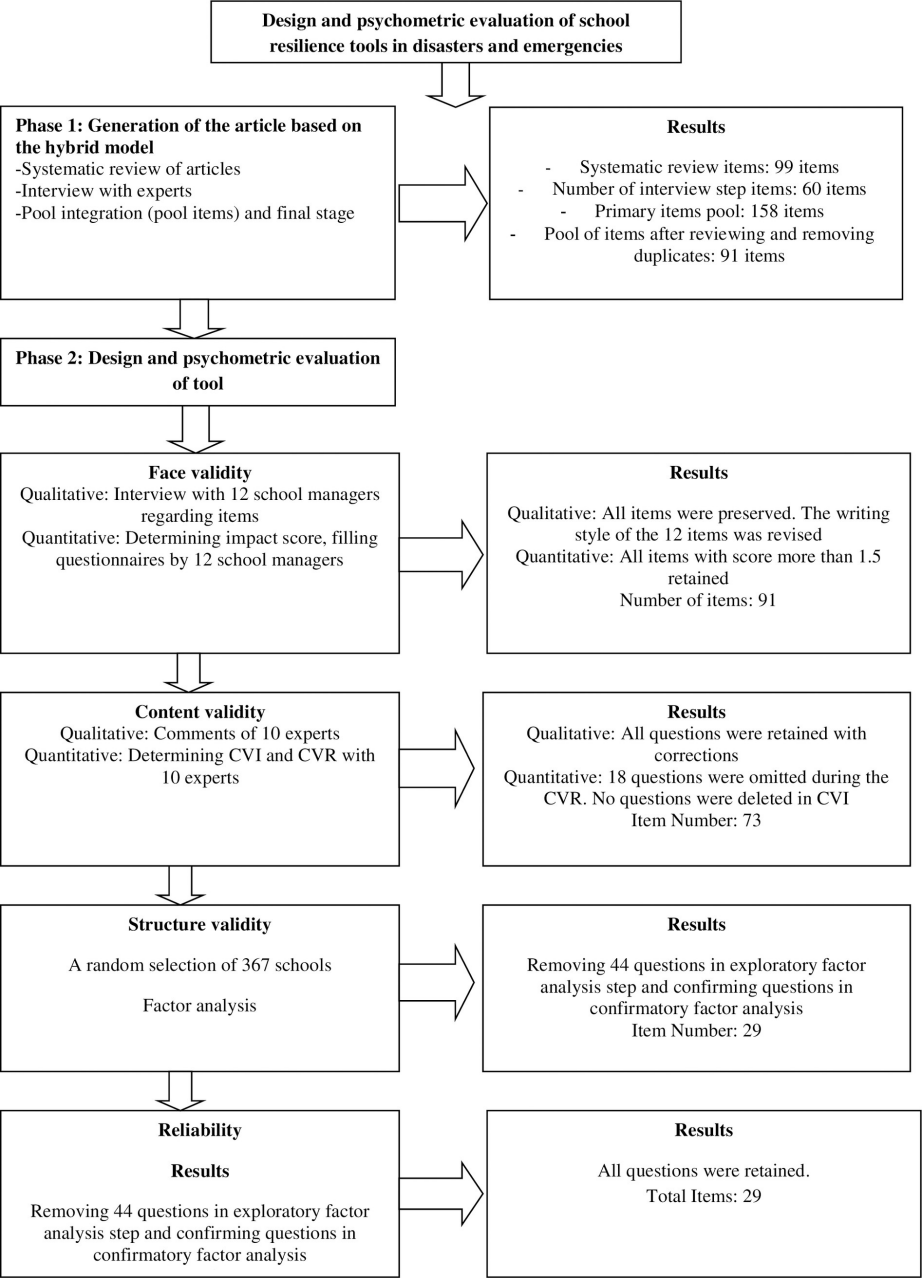
Production phases of school resilience tools in disasters and emergencies.

The present study was conducted using a sequential exploratory composite approach. The research was conducted in two main phases of production of the items based on hybrid model [34] and psychometric instrument [35]. These phases have been shown in Fig 1.

### Item generation phase

In this phase, a hybrid model including three stages of systematic review, interview with experts and final analysis were used. Firstly, a systematic review of published articles on the components of school resilience was conducted without time limit until 2018 at PubMed, Scopus, Web of Science and Google Scholar databases with keywords of resilience, schools and disasters. A total of 31 articles were selected in the study after reviewing 8053 extracted articles [20].

In the second step, a semi-structured interview was conducted for 30–60 minutes with 24 professionals in the fields of emergencies and disaster, structural engineers, psychologists, teachers and executive managers identifying resilient schools operating strategies until the data were finally analyzed according to the steps proposed by Graneheim and Lundman [22].

In the third stage, the components obtained in two stages were merged and after eliminating duplicates based on the class and subcategories of the previous stage, the pool of items was generated. At this stage, the preliminary tool was made after the initial review in the research group. For each item, a very low to very high response scale was considered based on a 5-point Likert scale.

### Phase of reduction item

It included validity and reliability.

The formal validity of the questionnaire was assessed by both qualitative and quantitative methods.

**In the qualitative formal validity,** 12 managers; teachers or assistants of the school, provided with questionnaires. Then, their views on appropriateness, difficulty, relevance and ambiguity of the questions were collected, and necessary corrections were made. The time required to respond to the tool was also estimated.

**On the other hand, the quantitative formal validity** comprised of same 12 managers; teachers or assistants of the school who were asked to state the importance of each item using a 5-point Likert scale from quite important (score 5) to no matter (score 1). Then, the important score of each tool item was calculated by examining the item impact using the formula of frequency × importance. In the item impact method, if the score is equal to or greater than 1.5, the item is identified appropriate and maintained for subsequent analysis [36].

### Content validity

The quantitative and qualitative methods were used to determine content validity [37].

At this stage, a questionnaire on qualitative content validity was sent to 10 experts with knowledge and experience in tool design, emergencies and disasters, health education, structural engineers and education chiefs. They were asked to provide the necessary feedback after qualitative evaluation of the questionnaire based on criteria of grammar, using appropriate words, placement of phrases in their proper places, and their corrective comments were applied in the questionnaire after discussion in research group.

**In the quantitative content validity,** two indices of content validity index (CVR) and item content validity index (CVI) were used to assess the quantitative content validity.

Initially, to determine CVR, the questionnaire was sent to 10 experts to examine each item based on a three-part Likert scale (useful but not necessary). CVR was calculated based on formula = (ne–(N/2))/ (N/2). In this formula, N was equal to the total number of specialists, and ne was the number of experts who gave the score of "necessary". According to the Lawshe’s table [38] and the number of 10 experts, items with a score below 0.62 were omitted to determine the minimum CVR.

Also, the content validity index of Waltz and Basel [39] was used to determine the relevance of each item of the questionnaire. For this purpose, 10 experts were asked to examine the items relevance based on a 4-point Likert scale of 1: Not relevant at all, 2: Somewhat relevant, 3: Relatively relevant, 4: Fully relevant. Content validity index score (CVI) for each item was calculated by dividing the number of experts who selected options of relevant and fully relevant for items (scores 3 and 4) by the total number of experts [40]. If the score for each item was greater than 0.79, it remained in the questionnaire and if the CVI score was less than 0.79, it was excluded [41].

### Construction validity

Exploratory and confirmatory factor analyses were used to determine the construction validity. In the first step, Exploratory Factor Analysis (EFA) was performed by extracting hidden factors using maximum likelihood (ML), using promax rotation, using SPSS_22_. The questionnaire was completed by 400 people, including the managers, teachers or assistants of school. Sample size in this step was determined based on at least 5 samples for each item designed in the questionnaire considering the number of questions [42] and 10% probability of falling. The suitability of the data was evaluated by exploratory factor analysis with two tests of evaluating sampling quality of KMO (Kaiser-mayer-olkin) and Bartlett’s spherical test. Values above 0.8 were considered appropriate [42]. To obtain the most desirable number of factors of total variance expressed, factor Eigen value and factor loading pattern were investigated. Items with a factor loading equal to or greater than 0.3 were considered appropriate and also specific values of one or less were not considered [43].

In the second step, the extracted factors were evaluated by confirmatory factor analysis (CFA) and the most common goodness-of-fit indices based on accepted threshold were estimated by AMOS software. Jaccard and Wan (1996) have been expressed Chi-square goodness of fit index, Root Mean Square Error of Approximation (RMSEA), Target Comparative Fit Index(CFI), Normed Fit Index (NFI), Adjusted Goodness of Fit Index (AGFI), TLI (Tucker-Lewis Index) and Parsimonious Comparative Fit Index (PCFI) and finally expressed chi-square ratio (χ^2^/df) as the most common goodness-of-fit index [44].

### Convergent and divergent validity

Convergent and divergent validities of the construction were measured by evaluating Average Variance Extract (AVE), Maximum Shared Squared Variance (MSV) and Average Shared Square Variance (ASV). AVE must be greater than 5.0 for convergent validity, AVE must be lower than MSV, and ASV for divergent validity [45–47].

### Tool reliability

Internal similarity and stability were used to determine the reliability of the instrument. To determine the internal similarity, the correlation coefficients of the questions in each dimension and the whole questionnaire was calculated using Cronbach’s alpha (α), Omega McDonald’s (Ω) and average inter-item correlation (AIC) [48]. However, alpha values above 0.7 are generally considered a sign of acceptable tool reliability [49], some researchers found alpha values between 0.6 and 0.9 to be appropriate depending on the nature of the tools and constructions measured [50] In order to investigate the stability of the tool over time (test-retest), the intra-class correlation index (ICC) was used and estimated at 0.95% confidence interval. For this purpose, the tool designed by a sample of 30 school managers was completed in two steps with a two-week interval. If this index is above 0.75, the stability is considered optimal [51]. Omega McDonald’s coefficient is calculated based on Ω = 1-[[a-Σhi]/[a+2b]] where a is the number of factor questions, Σhi is the subscription of the items or the sum of communality, and b is the sum of the factor loading that items, the Omega coefficient is between zero and one [52].

### Ethical considerations

This study is the result of a doctoral dissertation on health emergencies and disasters approved by the Ethics Committee of Yazd University of Medical Sciences with ethics code; IR.SSU.SPH.REC.1397.046. Prior to completing the questionnaires, the participants (school managers, teachers and assistants) were given a detailed explanation on the aims of the study and entered with consent. Informed written consent was obtained from each participant, and they were given the right to withdraw at any stage of the study. All participants were assured that their information would be kept confidential.

## Results

In the first phase, systematic review of 31 articles, 99 codes were extracted and classified as 4 themes of structural, non-structural, functional facility with 11 sub-themes including building standards, school premises, physical safety of buildings, facilities and equipment, safety and renovation of non-structural component, infrastructure, communications (internal and external), education, management, health and human-financial resources [20]

In the qualitative phase, 60 codes were extracted from interviews conducted with experts in four main categories including structural and non- structural improvement, improving organizational coordination and interactions, improving training and process, improving structural risk management subcategories and physical construction of building, correct placement of construction, improving non- structural safety inter- organizational communication and external organizational communication, conducting family, students, managers and personal training courses. Conducting simulated practices, increasing preparedness, proper planning, creating or organizational construction and facilitating rehabilitation as operational strategies for creating resilient schools [21].

Then, in the third step of the first phase, by integrating the items of two systematic and qualitative stages, the preparatory tool containing 158 items was formulated and the number of items was reduced to 91 after the initial study in the research group. Then, the questionnaire was designed using these items with 10 main categories in different dimensions including location, structural, non-structural architecture, equipment, health and welfare services, communication, training, coordination, internal- and external organizational communication and functional.

In the second phase of the study, the first step of formal validity was corrected according to the opinions of 12 school managers on how to write the 12 items, and since all items had an impact scored greater than 1.5, no statement was omitted at this stage.

In the content validity phase, 18 out of 91 items that have CVR less than 0.6 were deleted and the total number was reduced to 73. At the CVI examination stage, none of the items scored below 0.79 and no items were excluded. The mean content validity index was estimated to be 0.71 based on the mean content validity index scores of all questionnaire items.

8.25% of the 400 questionnaires were omitted due to incompleteness and 367 (91.75%) questionnaires were completed and received, and data of 367 questionnaires were analyzed. Participants in this study were 238 (64.9%) teachers, 35 (9.5%) school assistants and 94 (25.6%) managers with a mean age of 40.35±8.62 years with a work experience of 17.64±7.3 years. 196 (53.4%) subjects were female and 95 (25.9%) subjects had postgraduate education while 261 (71.1%) subjects had bachelor degrees and the rest had diplomas. 151 (41.14%) of them experienced natural hazards such as flood, fire and drought, and 149 (40.6%) had no experience of emergencies or disasters during their service.

Sampling adequacy index (KMO) was 0.89 and Bartlett’s Test of Sphericity was X2 = 6989.68 (p <0.001). In exploratory factor analysis, 5 factors (functional, architecture, education, safety and equipment) were extracted. These 5 factors had specific values of 5.763, 2.861, 2.690, 2.054, 1.741 and 52.08% of the total variance of variables of questionnaire of the school resilience assessment explained. Using the inflection point of 0.3 as the minimum factor load required to retain the item in the factors extracted from analysis, exploratory factor analysis was performed using principal component determination method and 44 questions were omitted at this stage and 29 remained in this step (Table 1). Confirmatory factor analysis was performed on the items to confirm the data and the questionnaire with 29 questions and 5 factors were approved.

**Table 1:** Exploratory factors extracted from the questionnaire of school resilience in emergencies and disasters.

special value	Percentage of variance	Subscription of items	Factor load	Item	Factor Name	Factor
5.763	19.87%	0.659	0.880	69. Contracts and agreements on coordination between the school, other organizations and local authorities for disasters and emergencies have been concluded.	Functional	First
		0.753	0.849	71. Stakeholders (Police, Firefighting, School Committee, Parents, Education and training, etc.) communication program and responsibilities have been specified in the school disaster preparedness program.		
		0.730	0.825	72. Students’ and parents’ opinions were used in developing disaster preparedness plans.		
		0.546	0.796	68. There is a process for informing parents when school events occur		
		0.501	0.795	70. Efforts have been made to involve donors and investors in the field of school restructuring and retrofitting.		
		0.699	0.763	73. Inter-school cooperation agreement with institutions or organizations providing psychological support to students and parents for post-disaster has been concluded		
		0.489	0.717	60. Speedway and crosswalk have been intended for pedestrians on the main path to the school exit door.		
		0.659	0.663	67. Depending on the expertise of the parents, coordination with them should be provided if assistance is needed in the time of disasters and emergencies.		
		0.637	0.583	51. There is a plan for unexpected events and events at school.		
		0.512	0.524	46. School staff, fire department and neighborhood governor are aware of the physical map and geographical situation of the school/neighborhood.		
		0.461	0.414	58. A list of hazardous chemicals in areas such as laboratories or warehouses has been provided		
2.861	9.86%	0.681	0.891	14. Distance and height of the window from the floor of the class and corridors are appropriate (minimum of 112 cm)	Architecture	Second
		0.515	0.705	17. Stairs height and width were suitable (maximum height of 18 cm and minimum width of 30 cm)		
		0.440	0.610	13. Standard space for each student in class has been considered (1.5 m on average)		
		0.429	0.599	18. Stairs and promontory areas had a tall and protective fence		
		0.474	0.589	20. Class doors were wide enough (80 cm)		
		0.283	0.501	21. Class doors opened out easily		
		0.328	0.492	8. Upgrading and modifying school facilities (heating, cooling, electricity and air-condition, water and sanitation systems) by experts.		
2.690	9.27%	0.759	0.895	38. Teachers and staff of school have passed first aid and rescue courses	Education	Third
		0.726	0.852	39. Managers, assistants and teachers were trained on appropriate measures for psychological support in disasters and emergencies.		
		0.740	0.835	37. Teachers and school staff have been trained on how to use a fire extinguisher and safety tips at school.		
		0.692	0.683	36. Managers, assistants, teachers and other school staff were trained disaster preparedness		
2.054	7.08%	0.818	0.914	24. There is an automatic fire alarm system in the school	Safety	Fourth
		0.721	0.842	25. The school fire alarm system is active		
		0.433	0.544	29. Anti-fire doors exist between hazardous school spaces such as laboratories and other parts of the building.		
		0.332	0.463	22. Plastic glass is used instead on top of the doors or glass is removed (Class door without glass inscription)		
1.741	6%	0.736	0.903	28. Fire extinguishers have been installed in sensitive locations on the wall and are easily accessible.	Equipment	Fifth
		0.700	0.846	27. All fire extinguishers are rechargeable and have a valid history		
		0.431	0.459	26. Fire control equipment such as fire extinguishers, sand bags, water access and hoses in available at school.		

In confirmatory factor analysis, chi-square goodness of fit test results were obtained [χ2 401, N = 367) = 1043.327, p < .001]. Then, other indices were evaluated to fit the model. According to acceptable level of indices, the appropriate fit of the final model was confirmed (Table 2).

**Table 2:** Fit indicators of confirmatory factor analysis model of assessment questionnaire of school resilience in emergencies and disasters.

IFI	AGFI	PNFI	PCFI	RMSEA	CMIN/DF	P-value	df	χ^2^	Fit Indicators * Confirmatory factor analysis model
0.901	0.813	0.732	0.776	0.066	2.602	<0.001	401	1043.327	First order after construction modification

*: Acceptable values of Index of PNFI, PCFI, AGFI (>0.5), CFI, IFI (>0.9), RMSEA (<0.08), CMIN/DF (3 <Good, 5 <Acceptable

**Fig 2:**
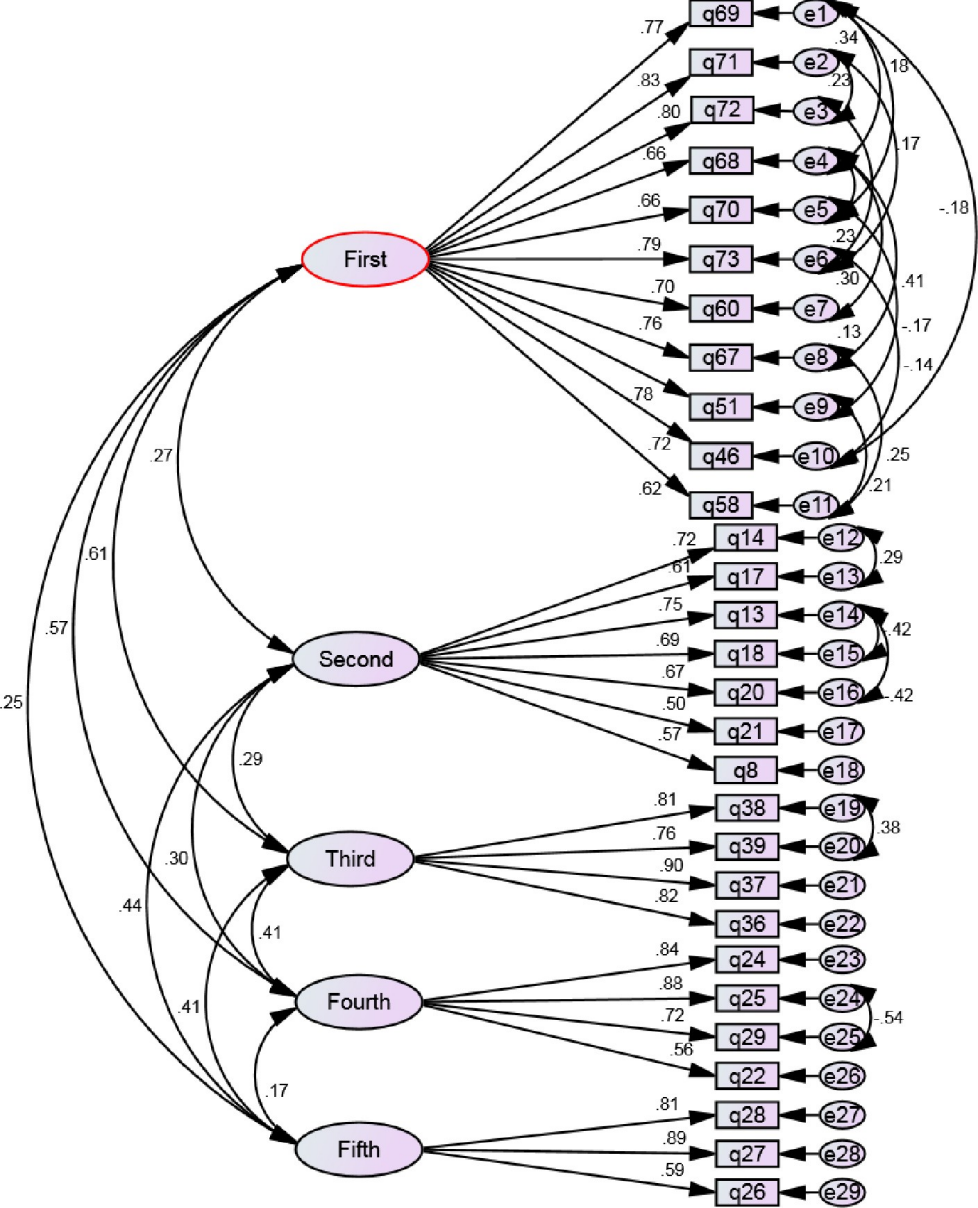
Constructions of evaluation of school resilience in emergencies and disasters: A modified confirmatory factor analysis model.

According to results presented in Table 3, the AVE of all factors is greater than 0.5, and the AVE of each factor is also greater than its MSV and CR is greater than AVE for all factors. The result showed that convergence and divergence validity of the school resilience assessment in emergencies and disasters is appropriate. The final modified model of confirmatory factor analysis of construction of school resilience assessment in emergencies and disasters has been shown in Fig 2.

**Table 3:** Convergent and divergent validity, internal stability and structural stability of questionnaire of evaluation of school resilience in emergencies and disasters.

CI,95%	ARC (Average inter-Item correlation)	McDonald’s Ω	Cronbach’s alpha	CR	MSV	AVE	Index Factor
Low-Upper							
0.895–0.923	0.594	0.912	0.910	0.929	0.373	0.544	First
0.769-.0832	0.404	0.807	0.802	0.836	0.230	0.425	Second
0.883–0.916	0.695	0.902	0.901	0.894	0.373	0.678	Third
0.784–0.845	0.526	0.824	0.816	0.843	0.320	0.579	Fourth
0.757–0.829	0.564	0.810	0.796	0.813	0.193	0.597	Fifth

Internal consistency of questions in questionnaire of school resilience assessment in emergencies and disasters was calculated at 0.95 using Cronbach’s alpha. Interclass correlation coefficient (ICC) was also obtained at 0.97 [95% confidence interval: 0.96–0.98]). Omega-McDonald’s coefficient and ARC (Average interitem correlation) were also reported for all factors. The ARC of the factors should be between 0.2 and 0.4 and values between 0.1 and 0.5 are acceptable [29]. In this study, the values were acceptable for all factors. Finally, construction reliability (CR) was calculated. CR was considered as an alternative to Cronbach’s alpha coefficient in structural equation model analysis. In the present study, CR more than 7% was considered acceptable [29].

## Discussion

This study was aimed to design and psychometric evaluation of schools’ resilience tool in Emergencies and disasters by a mixed-method. After the systematic [20] and quality study [21], the first primary schools’ resilience tool designed and fallowed the psychometrics process. The final school resilience in emergencies and disasters tool included: 29 questions with 5 dimensions; functional (11), architecture (7), education (4), equipment (3) and safety (4) that explained 52.08% of the total variance. All the dimensions of resilience were positively correlated with each other.

Based on the findings of the study, one of the dimensions for measuring the resilience of schools is functional. In this regard, Thi et al (2012) [53] and Shiwaku et al (2016) [18], also referred to it as one of school resilience and Grimaz et al(2016) [19] Considered the functional dimension as an important in the school safety besides others such as structural, non-structural and school location. Things like developing school disaster preparedness plans, providing the necessary equipment for emergency response, school and neighborhood mapping, identifying disaster management organizations, preparing phone lists, setting up school emergency alert systems reported for increasing school readiness and proper performance to respond to disasters [22].

Another dimension of this questionnaire is the architectural that is important for measuring school resilience in emergencies and disasters [20]. The architectural and structural features of school buildings are important issues in resilience which preserve the performance of schools after disasters and, most importantly, protect the lives of students [21]. Some of these have been mentioned in other tools for assessing school resilience under physical conditions of building and shape of the school [22].

The third dimension of designed tool is education. Thi (2012) [53] Stated education and evaluating disaster and emergencies, knowledge of students and teachers to assess school resilience as a component of human resources, its sub-component. Disasters and emergencies training courses and simulated exercises for students, teachers and families have been expressed to increase the resilience of schools [22].

Other dimensions of the questionnaire include safety and equipment. The existence of firefighting and fire safety equipment is one of the most important measures for measuring school resilience. Hosseini (2005) [54] recommended formation of fire brigade teams and training and equipping schools with fire extinguishers. The study of Hasnain (2006) [51] has suggested reparation and safety guidance against fire in addition to preparing fire extinguishers, and if there is no chance of extinguishing the fire, it is recommended to teach how to leave the school [20].

The reliability test of the tool showed internal consistency. The values of alpha, ARC (Average Inter-Item Correlation) and omega were accepted for each tool factor. In this study, all equivalent indices of model fit were evaluated and confirmatory factor results showed acceptable fitness of model.

According to CFA in the confirmatory factor model, the questionnaire with 5 dimensions and 29 questions is a suitable tool with acceptable validity and reliability for measuring school resilience in disasters and emergencies.

Alpha Cronbach’s School Resilience Assessment Questionnaire is 0.95. There have been no studies that have introduced the disaster resilience tools for schools [13, 50, 53]. Each of the dimensions of the questionnaire including functional (α = 0.910), architecture (α = 0.802), education (α = 0.901), equipment (α = 0.816), safety (α = 0.796) have been reported. Considering the acceptable reliability of all dimensions of this questionnaire, it can be used in measuring school resilience in emergencies and disasters. On the other hand, checking the ICC, reporting on the whole questionnaire and all dimensions are the benefits of this questionnaire.

## Conclusion

This study developed a questionnaire contacting 29-item for assessing of the school resilience in Persian language. The School Resilience Assessment Questionnaire is valid (face, content, and construct validity) and reliable (Alpha Cronbach, CR) questionnaire developed for assessment of school resilience in emergencies and disasters. This questioner is suitable be used in disaster and research settings to quantify school resilience. The Disaster Resilience Assessment Questionnaire provides the opportunity to intervene for improvement in times of emergencies and disasters, including disaster risk reduction programs that should be addressed by governments, especially in high-risk countries. Improving and increasing each aspect of functional education, safety, architecture, location and equipment will also increase disaster resiliency levels in schools. Although it has done in Iran; a country in Asia; other study in different country might figure out another criteria. Whole over, this primary schools’ resilience tool (School Resilience Assessment Questionnaire) in Emergencies and disasters with 29Q would be a basic for future study in different country.

## Supporting information

S1 Fig Production phases of school resilience tools in disasters and emergencies. (TIFF)

S2 Fig Constructions of evaluation of school resilience in emergencies and disasters: A modified confirmatory factor analysis model. (TIFF)

S1 Table Exploratory factors extracted from the questionnaire of school resilience in emergencies and disasters. (DOC)

S2 Table Fit indicators of confirmatory factor analysis model of assessment questionnaire of school resilience in emergencies and disasters. (DOC)

S3 Table Convergent and divergent validity, internal stability and structural stability of questionnaire of evaluation of school resilience in emergencies and disasters. (DOC)

S1 File (DOC)

S1 Data (XLSX)

## Abbreviations

CVR: content validity index
CVI: content validity index
EFA: Exploratory Factor Analysis
ML: Maximum Likelihood
KMO: Kaiser- Mayer- Olkin
CFA: Confirmatory Factor Analysis
RMSEA: Root Mean Square Error of Approximation
NFI: Normed Fit Index
CFI: Comparative Fit Index
AGFI: Adjusted Goodness of Fit Index
TLI: Tucker-Lewis Index
PCFI: Parsimonious Comparative Fit Index
AVE: Average Variance Extract
MSV: Maximum Shared Squared Variance
ASV: Average Shared Square Variance
AIC: Average Inter-item Correlation
ICC: Intra-class Correlation Index
ARC: Average inter item correlation
CR: Construction Reliability
