# Supplementary figures and images for: Design and psychometric evaluation of schools’ resilience tool in Emergencies and disasters: A mixed-method
Source: PLoS One. 2021 Jul 22;16(7):e0253906. doi: 10.1371/journal.pone.0253906 (PMC8297909; doi:10.1371/journal.pone.0253906)

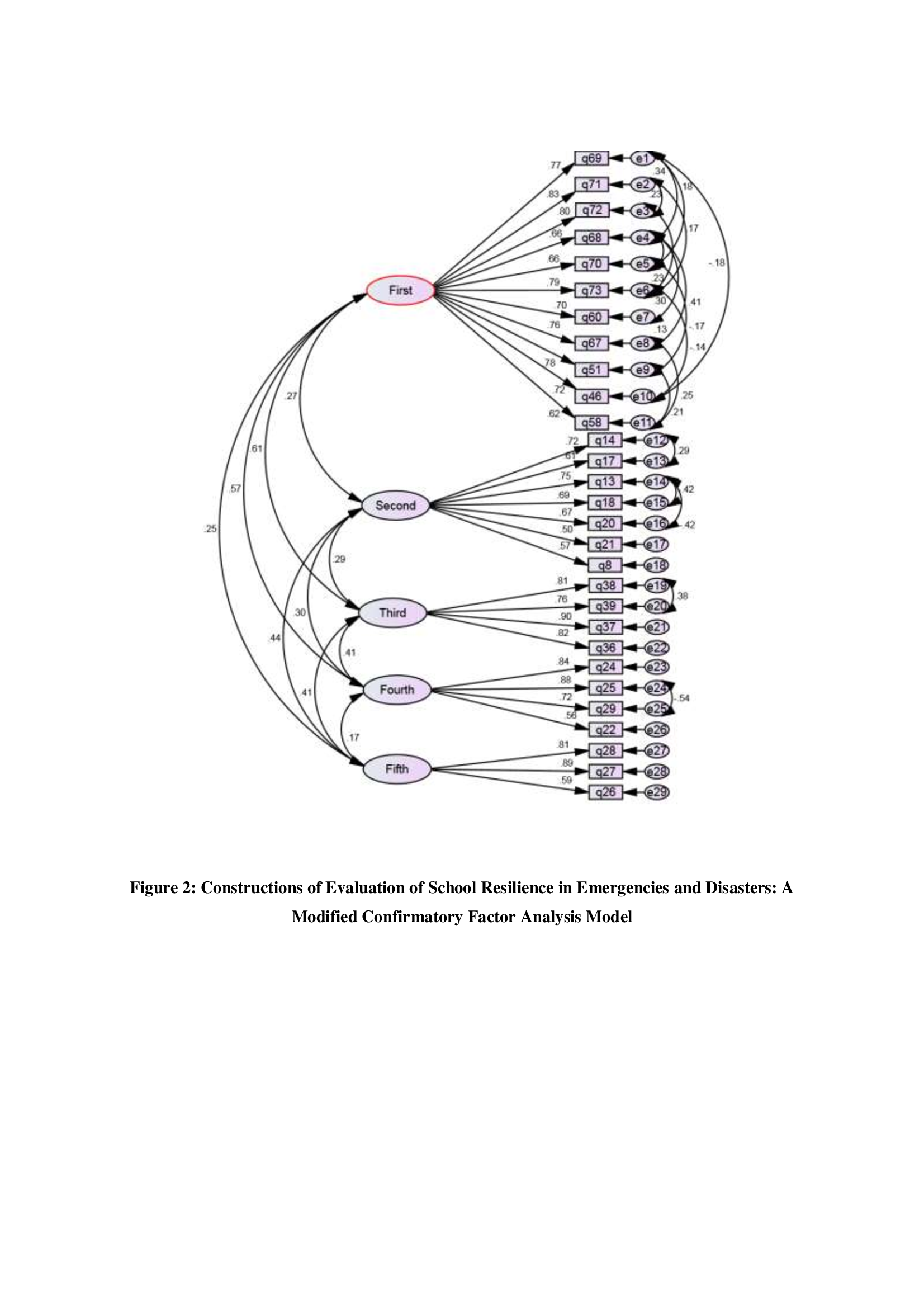

Supplement: S2 Fig — (TIFF) [file pone.0253906.s002.tiff]
