# Supplementary material for: Design and psychometric evaluation of schools’ resilience tool in Emergencies and disasters: A mixed-method
Source: PLoS One. 2021 Jul 22;16(7):e0253906. doi: 10.1371/journal.pone.0253906 (PMC8297909; doi:10.1371/journal.pone.0253906)
Supplement: S1 Table — (DOC) [file pone.0253906.s003.doc]

**S1 Table: Exploratory factors extracted from the Questionnaire of School Resilience in Emergencies and Disasters**

| **Factor** | **Factor Name** | **Item** | **Factor load** | **Subscription of items** | **Percentage of variance** | **special value** |
| --- | --- | --- | --- | --- | --- | --- |
| First | Functional | 69. Contracts and agreements on coordination between the school, other organizations and local authorities for disasters and emergencies have been concluded. | 0.880 | 0.659 | 19.87% | 5.763 |
|  |  | 71. Stakeholders (Police, Firefighting, School Committee, Parents, Education and training, etc.) communication program and responsibilities have been specified in the school disaster preparedness program. | 0.849 | 0.753 |  |  |
|  |  | 72. Students' and parents' opinions were used in developing disaster preparedness plans. | 0.825 | 0.730 |  |  |
|  |  | 68. There is a process for informing parents when school events occur | 0.796 | 0.546 |  |  |
|  |  | 70. Efforts have been made to involve donors and investors in the field of school restructuring and retrofitting. | 0.795 | 0.501 |  |  |
|  |  | 73. Inter-school cooperation agreement with institutions or organizations providing psychological support to students and parents for post-disaster has been concluded | 0.763 | 0.699 |  |  |
|  |  | 60. Speedway and crosswalk have been intended for pedestrians on the main path to the school exit door. | 0.717 | 0.489 |  |  |
|  |  | 67. Depending on the expertise of the parents, coordination with them should be provided if assistance is needed in the time of disasters and emergencies. | 0.663 | 0.659 |  |  |
|  |  | 51. There is a plan for unexpected events and events at school. | 0.583 | 0.637 |  |  |
|  |  | 46. School staff, fire department and neighborhood governor are aware of the physical map and geographical situation of the school/neighborhood. | 0.524 | 0.512 |  |  |
|  |  | 58. A list of hazardous chemicals in areas such as laboratories or warehouses has been provided | 0.414 | 0.461 |  |  |
| Second | Architecture | 14. Distance and height of the window from the floor of the class and corridors are appropriate (minimum of 112 cm) | 0.891 | 0.681 | 9.86% | 2.861 |
|  |  | 17. Stairs height and width were suitable (maximum height of 18 cm and minimum width of 30 cm) | 0.705 | 0.515 |  |  |
|  |  | 13. Standard space for each student in class has been considered (1.5 m on average) | 0.610 | 0.440 |  |  |
|  |  | 18. Stairs and promontory areas had a tall and protective fence | 0.599 | 0.429 |  |  |
|  |  | 20. Class doors were wide enough (80 cm) | 0.589 | 0.474 |  |  |
|  |  | 21. Class doors opened out easily | 0.501 | 0.283 |  |  |
|  |  | 8. Upgrading and modifying school facilities (heating, cooling, electricity and air-condition, water and sanitation systems) by experts. | 0.492 | 0.328 |  |  |
| Third | Education | 38. Teachers and staff of school have passed first aid and rescue courses | 0.895 | 0.759 | 9.27% | 2.690 |
|  |  | 39. Managers, assistants and teachers were trained on appropriate measures for psychological support in disasters and emergencies. | 0.852 | 0.726 |  |  |
|  |  | 37. Teachers and school staff have been trained on how to use a fire extinguisher and safety tips at school. | 0.835 | 0.740 |  |  |
|  |  | 36. Managers, assistants, teachers and other school staff were trained disaster preparedness | 0.683 | 0.692 |  |  |
| Fourth | Safety | 24. There is an automatic fire alarm system in the school | 0.914 | 0.818 | 7.08% | 2.054 |
|  |  | 25. The school fire alarm system is active | 0.842 | 0.721 |  |  |
|  |  | 29. Anti-fire doors exist between hazardous school spaces such as laboratories and other parts of the building. | 0.544 | 0.433 |  |  |
|  |  | 22. Plastic glass is used instead on top of the doors or glass is removed (Class door without glass inscription) | 0.463 | 0.332 |  |  |
| Fifth | Equipment | 28. Fire extinguishers have been installed in sensitive locations on the wall and are easily accessible. | 0.903 | 0.736 | 6% | 1.741 |
|  |  | 27. All fire extinguishers are rechargeable and have a valid history | 0.846 | 0.700 |  |  |
|  |  | 26. Fire control equipment such as fire extinguishers, sand bags, water access and hoses in available at school. | 0.459 | 0.431 |  |  |
