# Supplementary material for: Design and psychometric evaluation of schools’ resilience tool in Emergencies and disasters: A mixed-method
Source: PLoS One. 2021 Jul 22;16(7):e0253906. doi: 10.1371/journal.pone.0253906 (PMC8297909; doi:10.1371/journal.pone.0253906)
Supplement: S2 Table — (DOC) [file pone.0253906.s004.doc]

**S2 Table: Fit Indicators of Confirmatory Factor Analysis Model of Assessment Questionnaire of School Resilience in Emergencies and Disasters.**

| **IFI** | **AGFI** | **PNFI** | **PCFI** | **RMSEA** | **CMIN/DF** | **P-value** | **df** | **χ^2^** | Fit Indicators *  Confirmatory factor analysis model |
| --- | --- | --- | --- | --- | --- | --- | --- | --- | --- |
| 0.901 | 0.813 | 0.732 | 0.776 | 0.066 | 2.602 | <0.001 | 401 | 1043.327 | First order after construction modification |

*: Acceptable values of Index of PNFI, PCFI, AGFI (>0.5), CFI, IFI (>0.9), RMSEA (<0.08), CMIN/DF (3 <Good, 5 <Acceptable
