# Supplementary material for: Design and psychometric evaluation of schools’ resilience tool in Emergencies and disasters: A mixed-method
Source: PLoS One. 2021 Jul 22;16(7):e0253906. doi: 10.1371/journal.pone.0253906 (PMC8297909; doi:10.1371/journal.pone.0253906)
Supplement: S3 Table — (DOC) [file pone.0253906.s005.doc]

**S3 Table: Convergent and Divergent Validity, Internal Stability and Structural Stability of Questionnaire of Evaluation of School Resilience in Emergencies and Disasters**

| **Index**  **Factor** | AVE | MSV | CR | Cronbach's alpha | McDonald's Ω | **ARC**  **(Average inter-Item correlation)** | **CI,95%**  **Low-Upper** |
| --- | --- | --- | --- | --- | --- | --- | --- |
| First | 0.544 | 0.373 | 0.929 | 0.910 | 0.912 | 0.594 | 0.895-0.923 |
| Second | 0.425 | 0.230 | 0.836 | 0.802 | 0.807 | 0.404 | 0.769-.0832 |
| Third | 0.678 | 0.373 | 0.894 | 0.901 | 0.902 | 0.695 | 0.883-0.916 |
| Fourth | 0.579 | 0.320 | 0.843 | 0.816 | 0.824 | 0.526 | 0.784-0.845 |
| Fifth | 0.597 | 0.193 | 0.813 | 0.796 | 0.810 | 0.564 | 0.757-0.829 |
