# Supplementary material for: Design and psychometric evaluation of schools’ resilience tool in Emergencies and disasters: A mixed-method
Source: PLoS One. 2021 Jul 22;16(7):e0253906. doi: 10.1371/journal.pone.0253906 (PMC8297909; doi:10.1371/journal.pone.0253906)
Supplement: S1 File — (DOC) [file pone.0253906.s006.doc]

| Developed **Questionnaire of School Resilience in Emergencies and Disasters**  by research | | | | | | | |
| --- | --- | --- | --- | --- | --- | --- | --- |
| **Very little** | **little** | **Some**  **what** | **much** | **very much** | questions | areas | N |
|  |  |  |  |  | Contracts and agreements on coordination between the school, other organizations and local authorities for disasters and emergencies have been concluded. | Functional | 1 |
|  |  |  |  |  | Stakeholders (Police, Firefighting, School Committee, Parents, Education and training, etc.) communication program and responsibilities have been specified in the school disaster preparedness program. |  | 2 |
|  |  |  |  |  | Students' and parents' opinions were used in developing disaster preparedness plans. |  | 3 |
|  |  |  |  |  | There is a process for informing parents when school events occur |  | 4 |
|  |  |  |  |  | Efforts have been made to involve donors and investors in the field of school restructuring and retrofitting. |  | 5 |
|  |  |  |  |  | Inter-school cooperation agreement with institutions or organizations providing psychological support to students and parents for post-disaster has been concluded |  | 6 |
|  |  |  |  |  | Speedway and crosswalk have been intended for pedestrians on the main path to the school exit door. |  | 7 |
|  |  |  |  |  | Depending on the expertise of the parents, coordination with them should be provided if assistance is needed in the time of disasters and emergencies. |  | 8 |
|  |  |  |  |  | There is a plan for unexpected events and events at school. |  | 9 |
|  |  |  |  |  | School staff, fire department and neighborhood governor are aware of the physical map and geographical situation of the school/neighborhood. |  | 10 |
|  |  |  |  |  | A list of hazardous chemicals in areas such as laboratories or warehouses has been provided |  | 11 |
|  |  |  |  |  | Distance and height of the window from the floor of the class and corridors are appropriate (minimum of 112 cm) | Architecture | 12 |
|  |  |  |  |  | Stairs height and width were suitable (maximum height of 18 cm and minimum width of 30 cm) |  | 13 |
|  |  |  |  |  | Standard space for each student in class has been considered (1.5 m on average) |  | 14 |
|  |  |  |  |  | Stairs and promontory areas had a tall and protective fence |  | 15 |
|  |  |  |  |  | Class doors were wide enough (80 cm) |  | 16 |
|  |  |  |  |  | Class doors opened out easily |  | 17 |
|  |  |  |  |  | Upgrading and modifying school facilities (heating, cooling, electricity and air-condition, water and sanitation systems) by experts. |  | 18 |
|  |  |  |  |  | Teachers and staff of school have passed first aid and rescue courses | Education | 19 |
|  |  |  |  |  | Managers, assistants and teachers were trained on appropriate measures for psychological support in disasters and emergencies. |  | 20 |
|  |  |  |  |  | Teachers and school staff have been trained on how to use a fire extinguisher and safety tips at school. |  | 21 |
|  |  |  |  |  | Managers, assistants, teachers and other school staff were trained disaster preparedness |  | 22 |
|  |  |  |  |  | There is an automatic fire alarm system in the school | Safety | 23 |
|  |  |  |  |  | The school fire alarm system is active |  | 24 |
|  |  |  |  |  | Anti-fire doors exist between hazardous school spaces such as laboratories and other parts of the building. |  | 25 |
|  |  |  |  |  | Plastic glass is used instead on top of the doors or glass is removed (Class door without glass inscription) |  | 26 |
|  |  |  |  |  | Fire extinguishers have been installed in sensitive locations on the wall and are easily accessible. | Equipment | 27 |
|  |  |  |  |  | All fire extinguishers are rechargeable and have a valid history |  | 28 |
|  |  |  |  |  | Fire control equipment such as fire extinguishers, sand bags, water access and hoses in available at school. |  | 29 |
